# Distinct fusion intersegmental parameters regarding local sagittal balance provide similar clinical outcomes: a comparative study of minimally invasive versus open transforaminal lumbar interbody fusion

**DOI:** 10.1186/s12893-020-00765-0

**Published:** 2020-05-12

**Authors:** Fuping Li, Chen Li, Xin Xi, Zhili Zeng, Bin Ma, Ning Xie, Hang Wang, Yan Yu, Liming Cheng

**Affiliations:** 1 Department of Spine Surgery, Tongji Hospital, Tongji University School of Medicine, Shanghai, 200065 China; 2 Department of Orthopaedics, Jinghong People’s Hospital, Jinghong City, 666100 Yunnan Province China

**Keywords:** Transforaminal lumbar interbody fusion, Minimally invasion, Open, Fusion level, Sagittal balance, Clinical outcome

## Abstract

**Background:**

Most contemporary studies suggested that intersegmental parameters including disc height and local lordosis contribute to the sagittal balance of fused lumbar. Although similar clinical outcomes following MIS- and Open-TLIF were reported essentially at the early postoperative time, the comparison of local balance variables after these two different techniques was lack. The radiological differences maybe not relevant to the postoperative efficacy at an earlier post-operation stage. But during the long-term follow-up, the complications with regards to the sagittal imbalance might occur due to the distinct biomechanical properties of fusion level after MIS- and Open-TLIF.

**Methods:**

The patients who underwent a single-level MIS- and Open-TLIF were reviewed retrospectively. The anterior disc height (ADH), posterior disc height (PDH), and segmental lordosis (SL) of the fusion segment were measured using recognition technical fluoroscopy. The mean disc height (MDH) was calculated by (ADH + PDH)/2. The relative DH was normalized by the anterior height of the upper vertebrae. The body mass index (BMI), the pain score of low back and leg visual analogue scale (VAS), Oswestry disability index (ODI), estimated blood loss, and hospital stay length was collected.

**Results:**

A total of 88 patients undergoing a single-level TLIF (MIS and Open) were included. The pre- and post-operative ADH, PDH, MDH, and SL of MIS-TLIF group were 1.57 ± 0.33 cm, 0.79 ± 0.20 cm, 1.18 ± 0.21 cm, 7.36 ± 3.07 and 1.63 ± 0.30 cm, 1.02 ± 0.28 cm, 1.32 ± 0.24 cm, 10.24 ± 4.79 respectively. Whereas, the pre- and post-operative ADH, PDH, MDH, and SL of Open-TLIF group were 1.61 ± 0.40 cm, 0.77 ± 0.21 cm, 1.19 ± 0.24 cm, 9.05 ± 5.48 and 1.81 ± 0.33 cm, 0.98 ± 0.24 cm, 1.39 ± 0.24 cm, 12.34 ± 4,74 respectively. MIS- and Open-TLIF group showed no significant differences in low back VAS, leg VAS, and ODI both in pre-operation and post-operation (*P* > 0.05). The estimated blood loss and hospital stay length in the MIS-TLIF group were significantly lower than those in the Open-TLIF group (*P* < 0.05).

**Conclusion:**

MIS- and Open-TLIF provided similar clinical outcomes as the respect of low back VAS, leg VAS, and ODI. MIS-TLIF significantly reduced the blood loss and length of hospital stay though. The intervertebral parameters of DH and SL were both increased significantly, Open-TLIF group presented better sagittal balance in term of ADH and SL variables. The contrast investigation of intersegmental parameters may help the surgeons to figure out the further advantages of MIS-TLIF technique, and then better manage the rehabilitation and prevent the reoperation.

## Background

The lumbar intervertebral fusion technique is the gold standard for the treatment of lumbar disc degenerative diseases (DDD) for which conservative treatment is ineffective. Since the less violation of bony structure and spinal nerve, the TLIF technique is currently widely used worldwide as its advantage is better physiological lordosis, sagittal balance and fewer complications [1, 2]. Followed by the development of minimal instruments in the market [3], MIS-TLIF formed an innovative surgery for the treatment of lumbar DDD [1]. The main differences between the two are that MIS-TLIF reduces the area of the surgical incision and the damage to the paraspinal muscle tissue, and performs better in reducing surgical bleeding and reducing the risk of paraspinal muscle atrophy [4–6]. MIS-TLIF limits the space of operating for spine surgeons, which requires a higher capability and skillset from them. As a result, there is a prolonged operation time, an increased number of intraoperative fluoroscopies, and an increase in the risks of surgery up to 31.37% [7]. Although most current studies suggest that MIS and Open have similar effects in the early and middle post-operation stage [6, 8–10], some complications may occur due to the long-term effects of differences in biomechanical properties, such as adjacent segment disease (ASD) [11]. Once it occurs, it seriously affects the quality of patients’ lives. In severe cases, the patient needs to be operated on again, and the fusion of adjacent segments may be worse than in the first surgery [12], which brings added problems for spine surgeons. The reasons for the contradiction between minimal invasion and the outcomes may be relevant to the difference in imaging results that affect the post-operative immediate stability. The additional parameters regarding the operating site stability are supposed to be vital as well as the assessment of clinical outcomes [13].

Postoperative sagittal balance plays a more important role to maintain stability by biomechanical studies [13]. The balance variables, especially facet joint violation (FV), disc height and segmental lordosis angle, were controlled by the surgeons [14]. In our previous study, FV occurred at a higher incidence in MIS-TLIF that may result in ASD [15]. However, few studies have compared the other two parameters from these two surgeries. It is not clear whether it affects the outcome of surgery and long-term follow-up results.

In our study, we will analyze the restoration of intervertebral disc height and segment lordosis of the fusion segment after Open-TLIF and MIS-TLIF. We hypothesis that the fusion parameters present a significant difference, but the comparison of clinical outcomes would be improved similarly at follow-up. The investigation of intersegmental parameters may help the surgeons to figure out the patients’ selection for the MIS-TLIF technique other than classical Open-TLIF, and better manage the rehabilitation and prevent the reoperation subsequently.

## Materials and methods

### Follow-up method

Inclusion criteria: 1) Patients over 18 years of age; 2) agreed to a single-segment MIS-TLIF or Open-TLIF for the treatment of symptomatic lumbar degenerative diseases; 3) accomplished lumbar X-ray imaging before and after surgery, X-ray files were available and valid; 4) follow-up for at least 6 months.

Exclusion criteria: 1) Accepted Multi-segment surgery; 2) have previously performed open lumbar fusion surgery or not TLIF fusion, adjacent segment decompression, or other procedures, such as interspinous implant placement; 3) suffered lumbar deformity, infection, tumor, and trauma.

### Surgical procedure: bilateral open-TLIF and unilateral MIS-TLIF surgery were used in this research. The same sophisticated senior spine surgeon performed all procedures. All grafts were identical in type and from the same company

#### MIS-TLIF technique (Fig. 1)

Patients were in a prone position after general anesthesia. Four pedicles above and below the surgical segment were located under the perspective of the C-arm X-ray machine and then mark the skin. Then, puncture needles were inserted into four pedicles in turn. After inserting the guide needles and pulling out the puncture needles, a small incision about 4 cm long was made at the line between the upper and lower pedicle of the pathological side, then the skin and fascia were cut in turn. Bluntly separate the paraspinal muscle till the small facet joints; insert the Spotlight channel, and then connect the cold light illuminator. Peel off the paravertebral muscle with an electrosurgical generator to reveal the intervertebral space between the pedicles. After the removal of the fibrous ring and nucleus pulposus, the superior and inferior cartilage plates of the intervertebral space were also removed. The cut facet joint was used as a bone graft, partially placed in the cage, and the remaining part was implanted into the intervertebral space. The cage was then placed into the intervertebral space. Along the guide needle, the pedicle screw was tightened, and the connecting rod of the pedicle screw was placed and secured. After debridement and hemostasis, negative pressure drainage was placed, and the incision was sutured.

**Fig. 1:**
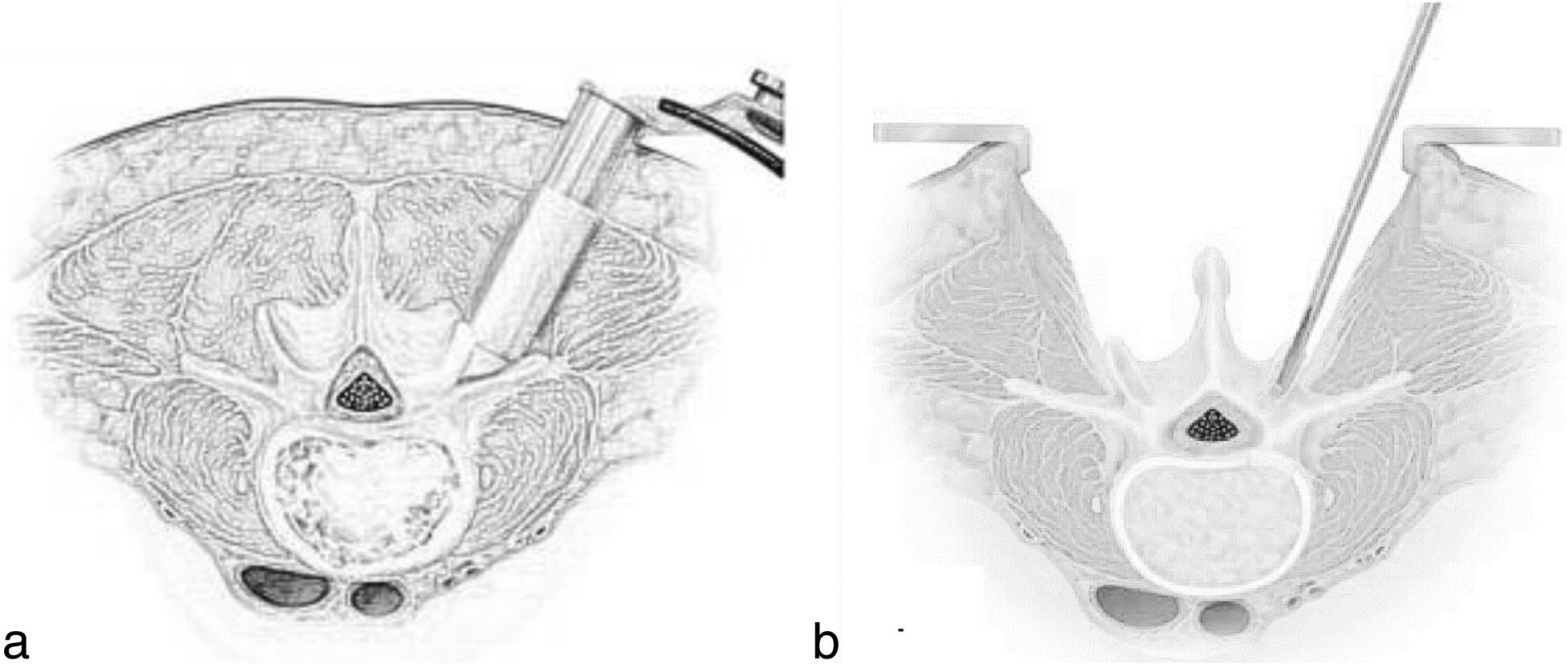
Surgerical procedure scheme: MIS-TLIF (**a**) and Open-TLIF (**b**) techniques

#### Open-TLIF technique (Fig. 1)

Patients were in a prone position after general anesthesia. Under the perspective of the C-arm X-ray machine, locate the segment to be operated on. The incision was made in the middle direction along the spinous process; skin and fascia were cut in turn, and then the paravertebral muscles were completely stripped till the facet joint by electrosurgical generator along the sidewall of the spinous process. Expose the facet joints and the transverse processes of the superior and inferior vertebral bodies and the lamina of the superior vertebral body. Laminectomy and decompression were performed with the laminar rongeur. The ligamentum flavum and facet joints were excised in turn. The intervertebral space was exposed, the nerve root and dura mater were protected, and the intervertebral space between pedicles was fully exposed. After removing the annulus fibrosus and the nucleus pulposus, the cartilage plate above and below the intervertebral space was removed. The cut facet joint before was used as a bone graft, partially placed in the cage, and the remaining part was implanted into the intervertebral space. The cage is then placed into the intervertebral space. Along the guide needle, the pedicle screw was tightened, and the connecting rod of the pedicle screw was placed and secured. After debridement and hemostasis, negative pressure drainage was placed, and the incision was sutured.

### Data collecting

**Fig. 2:**
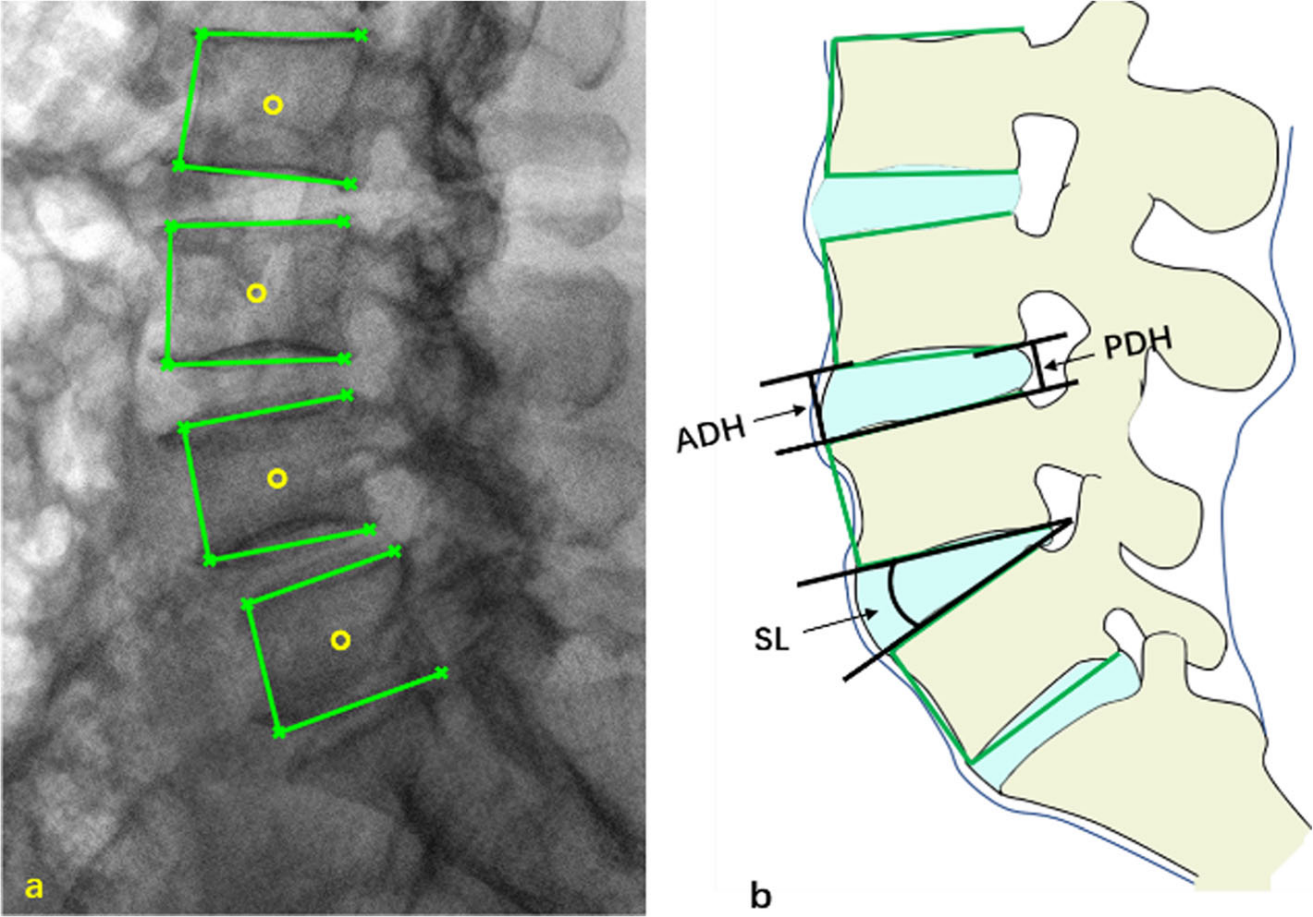
Parameter Measurement: a. recognition of vertebral margins via X-ray films; b. calculation of AHD, PDH and SL parameters

Preoperative and postoperative X-ray films of the patients undergoing TLIF or MIS-TLIF surgery for lumbar degenerative diseases between January 2013 and May 2016 were collected according to the inclusion and exclusion criteria. Anterior disc height (ADH), posterior disc height (PDH), and segmental lordosis (SL) was measured from the X-ray films based on the recognition technique [16–18] (Fig. 2). The MDH was calculated by (ADH + PDH)/2. The relative DH was normalized by the anterior height of the upper vertebrae. Functional results (including low back VAS, leg VAS, ODI, intraoperative blood loss, and length of hospital stay) were collected as well.

### Data processing

All data were imported into SPSS 21.0 (IBM, Armonk, New York, USA) for statistical analysis. Data were expressed as mean ± standard deviations for variables. Preoperative and postoperative differences were performed using a paired t-test. Open-TLIF and MIS-TLIF differences were performed using an independent t-test. Statistical significance was set as *P* < 0.05.

## Results

Our study included 88 patients. Fifty patients were in the Open-TLIF group including 35 lumbar spinal stenosis (LSS) and 15 lumbar disc herniation (LDH), male/female (25/25). The mean age was 54.42 ± 13.94 years old, and the mean body mass index was 25.78 ± 3.33 kg/m^2^. Thirty-eight patients were in the MIS-TLIF group including 24 LSS and 14 LDH, male/female (20/18). The mean age was 51.61 ± 12.33 years old, and the mean body mass index was 26.28 ± 3.23 kg/m^2^. Postoperative complications were not found in both groups in the follow-up of 6 months. Most patients (91%) had surgical segments at L4/5, L5/S1 levels. (Table 1).

**Table 1:** Patient information

	Open	MIS	P
Gender (M/F)	25/25	20/18	0.809
Age (yr.)	54.42 ± 13.94	51.61 ± 12.33	0.327
BMI (kg/m^2^)	25.78 ± 3.33	26.28 ± 3.23	0.473
Level of fusion			
L1/2	0	1	
L2/3	0	0	
L3/4	6	1	
L4/5	29	19	
L5/S1	15	17	

The values of ADH, PDH, MDH, and SL in the Open-TLIF group were 1.61 ± 0.40 cm, 0.77 ± 0.21 cm, 1.19 ± 0.24 cm, 9.05 ± 5.48 before operation and 1.81 ± 0.33 cm, 0.98 ± 0.24 cm, 1.39 ± 0.24 cm, 12.34 ± 4.74 after the operation, respectively. The values of ADH, PDH, MDH, and SL in the MIS-TLIF group were 1.57 ± 0.33 cm, 0.79 ± 0.20 cm, 1.18 ± 0.21 cm, 7.36 ± 3.07 before operation and 1.63 ± 0.30 cm, 1.02 ± 0.28 cm, 1.32 ± 0.24 cm, 10.24 ± 4.79 after the operation, respectively. Relative disc height was shown in Table 2.

**Table 2:** Relative Disc Height and Segmental Lordosis

	Open (*n* = 50)	MIS (*n* = 38)	P
Anterior Disc Height			
Preop	0.51 ± 0.12	0.49 ± 0.12	0.355
6 months postop	0.58 ± 0.11^*^	0.51 ± 0.10	0.002
Difference	0.07 ± 0.12	0.02 ± 0.11	0.050
Difference ratio (%)	18.54 ± 31.59	6.71 ± 22.28	0.053
Posterior Disc Height			
Preop	0.25 ± 0.06	0.25 ± 0.07	0.982
6 months postop	0.31 ± 0.08^*^	0.32 ± 0.10^*^	0.717
Difference	0.06 ± 0.09	0.07 ± 0.09	0.727
Difference ratio (%)	34.44 ± 47.20	34.64 ± 42.68	0.984
Mean Disc Height			
Preop	0.38 ± 0.8	0.37 ± 0.08	0.373
6 months postop	0.45 ± 0.08^*^	0.41 ± 0.09^*^	0.067
Difference	0.07 ± 0.09	0.05 ± 0.08	0.222
Difference ratio (%)	21.30 ± 32.73	14.35 ± 20.88	0.231
Segmental Lordosis (°)			
Preop	9.05 ± 5.63	7.36 ± 3.07	0.092
6 months postop	12.34 ± 4.74^*^	10.24 ± 4.79^*^	0.043
Difference	3.29 ± 5.71	2.88 ± 5.48	0.733

∗is significant differences in *p*-value(*p* < 0.05) between the first measurement and the second measurement

In Open-TLIF, the ADH, PDH, MDH, and SL significantly increased in 6 months post-operation (*p* < 0.05). In MIS-TLIF, the PDH, MDH, and SL significantly increased in 6 months post-operation (*p* < 0.05). The post-operation of ADH in the Open-TLIF group was significantly higher than that in MIS-TLIF (p < 0.05). However, there was no significant difference in PDH values between two surgeries (*p* > 0.05). The 6 months post-operation of SL in the Open-TLIF group was significantly higher than those in the MIS-TLIF group (p < 0.05). (Table 2).

**Fig. 3:**
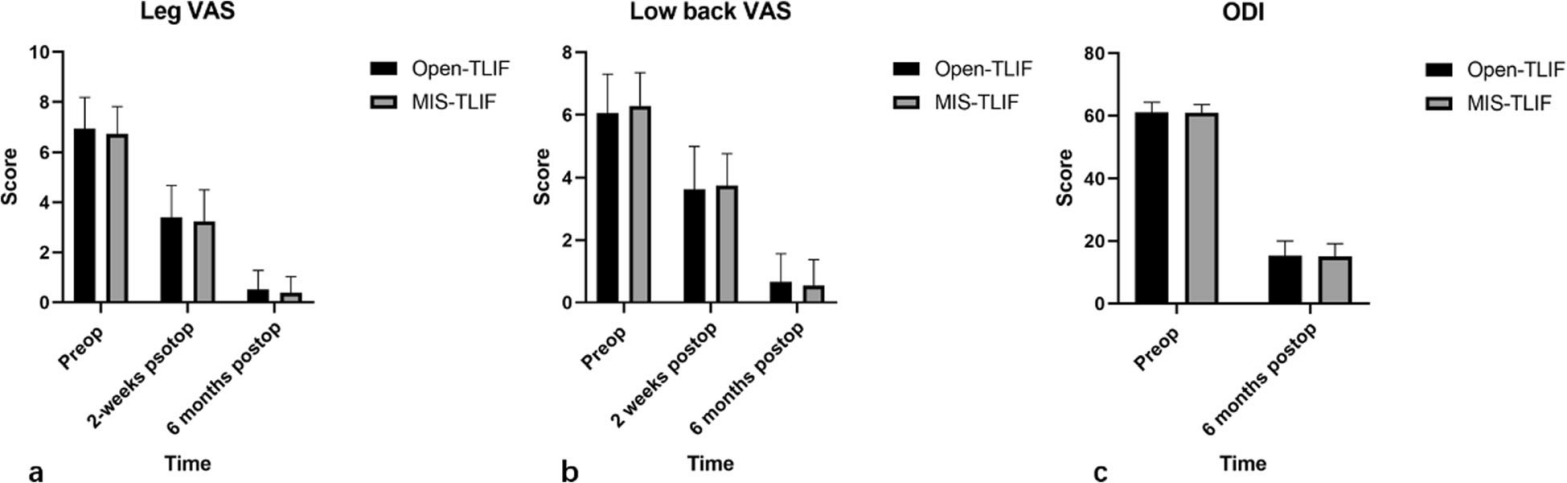
Functional scores showed no significant differences between MIS- and Open-TLIF techniques. **a**. Leg VAS; **b**. Low back VAS; c. ODI

**Fig. 4:**
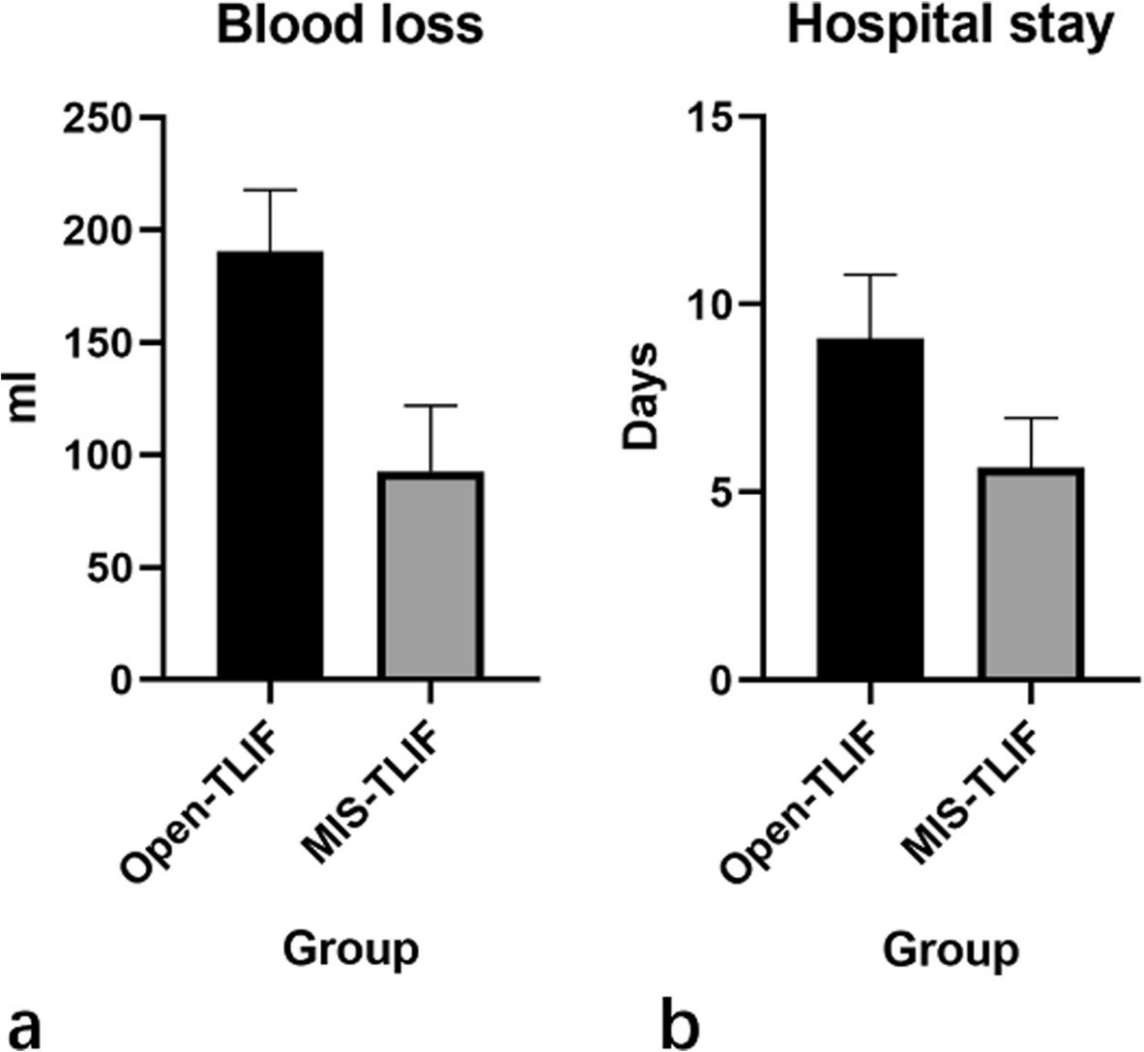
Significant differences about blood loss (**a**) and hospital stay(**b**) were presented between MIS- and Open-TLIF techniques

Open-TLIF and MIS-TLIF showed no significant differences in low back VAS, leg VAS, and ODI in pre-operation and post-operation (p > 0.05). However, the intraoperative blood loss and length of hospital stay of MIS-TLIF were significantly lower than those in the Open-TLIF group, and the difference was statistically significant (p < 0.05). (Figs. 3 & 4).

## Discussion

It is generally believed that MIS-TLIF is more advantageous, including less soft tissue damage, reduced blood loss, less postoperative pain, shorter hospital stays, and shorter recovery times, all the while, achieving clinical results comparable to those of an equivalent open surgery [5, 6, 8, 19]. However, MIS-TLIF also has some shortcomings [15, 20, 21], such as possible higher device-related complications, for example, cage misalignment, nerve root injury, and screw misplacement. There is also a need for more intraoperative fluoroscopy [22], resulting in increased radiation doses, which is unfriendly for patients and surgeons. At the same time, the higher requirement for spine surgeons is the prolonged study time [23]. Some studies have pointed out there to be improved methods to reduce fluoroscopy time, such as the use of image enhancement software combined with ultra-low-radiation imaging to achieve similar effects to standard-dose C-arm fluoroscopy [24], even with 3D navigation without fluoroscopy [25]. Considering the above factors, surgeons consistently struggled in dilemma when choosing between MIS- and Open-TLIF techniques.

Although the final clinical effects of MIS-TLIF and Open-TLIF were similar [20, 26, 27], the dedicated intersegmental parameters results have not been further analyzed. The surgical procedures are different that is speculated to be one of the main reasons for the differences in the DH and SL, such as the facet joint resection and the graft implantation. Compared with the Open-TLIF group, the MIS-TLIF group required a smaller incision area, less resected tissue, and less muscle damage, which may result in a smaller range of screw adjustment than the Open-TLIF group.

Biomechanically, the facet joint contributes to maintaining the stability of the lumbar spine, and it prevents two adjacent vertebrae from engaging in relative motions [28]. The MIS procedure only removes the facet joints on the decompression side, which may increase the resistance when expanding intervertebral space in the MIS surgery. In our study, follow-up radiological measurements were performed accurately using the recognition of postoperative X-ray. Both the Open-TLIF and MIS-TLIF groups increased intervertebral DH and SL post-operation, but the post-operation values of ADH and SL in the Open-TLIF group were higher than that in the MIS-TLIF. Pereira et al. [16] found that the intervertebral disc height had increased in the MIS-TLIF group after an operation. On the contrary, Kim et al. [18] found that the intervertebral disc height was increased significantly after an Open-TLIF operation, which is consistent with our results. Moreover, the procedure to place, adjust and fix the screw rod in MIS-TLIF is much more complicated than that in Open-TLIF procedures due to the minimally invasive incision. MIS-TLIF result in the smaller intervertebral SL accordingly. There were no significant changes in the SL values at fusion segments reported by Pereira et al., [16] it may be caused by the reserve of the opposite facet joint and the use of polyaxial screws.

The sagittal imbalance is consistently associated with adjacent segment disease after spinal fusion [29]. Due to solid fixation of fusion level, overloading of the adjacent segments may increase the risk of developing symptomatic ASD [30]. For patients undergone a lumbar spinal fusion, the preoperative sagittal imbalance may significantly increase the risk of ASD [31, 32]. Findings indicate that risks for complications might be reduced by the restoration of sagittal balance, appropriate deformity correction and advanced lumbosacral fixation. Restoration of sagittal balance can reduce the ASD [33]. However, other risk factors including age, genetic factors, high body mass index, pre-existing adjacent segment degeneration, smoking, corticosteroid use, etc. should also be considered and then the prevention strategy would be mandatory.

Some studies have suggested that MIS-TLIF is superior to Open-TLIF in reducing adjacent segment disease [34, 35]. The smaller damage caused by MIS-TLIF to the surrounding soft tissue and the recovery of postoperative spinal stability may contribute to this difference. However, recent studies suggest that SL affects the overall sagittal balance of the spine, adequate restoration of intervertebral disc height and SL is beneficial in reducing the degeneration of adjacent segments [36]. Poor restoration of intervertebral parameters by MIS-TLIF reported in our study influenced the sagittal balance and long-term structural stability. The improvement in surgical instruments and procedures is recommended. For instance, the utilization of expandable interbody fusion cages [37, 38] in MIS-TLIF is beneficial for restoring the intervertebral DH and SL without increasing associated complications. On the other hand, it’s necessary to unlock the bilateral facet joints during the MIS-TLIF procedure that helps to enlarge the fused disc space.

There’re some limitations to our study. Since the values of DH and SL immediately after the operation weren’t collected. The results of the follow-up at 6 months may be influenced by limited disc height restoration at the time of operation or subsidence in the follow-up period. We only focused on the local sagittal balance parameters including segmental lordosis and disc height of fusion level. Our study just enrolled in 6 months’ clinical and radiological outcomes. Further study on radiological parameters (e.g., global sagittal variables and spine-pelvic parameters) and long-term follow-up would be of great significance in the following research. We investigated the static parameters of human lumbar. In the future, in vivo parameters under functional motions (bending, extension, and rotation) and the most common loading condition during daily life should also be investigated.

## Conclusion

Open-TLIF and MIS-TLIF show similar clinical outcomes in low back VAS, leg VAS, and ODI, but MIS-TLIF significantly reduced the intraoperative blood loss and length of hospital stay. Both Open-TLIF and MIS-TLIF have increased the intervertebral DH and SL. The Open-TLIF is superior to MIS-TLIF to restore the local ADH and SL.

## Data Availability

The datasets generated and/or analyzed during the current study are not publicly available due to the data is confidential patient data but are available from the corresponding author on reasonable request.

## Abbreviations

MIS: minimally invasive
TLIF: transforaminal lumbar interbody fusion
ADH: anterior disc height
PDH: posterior disc height
MDH: mean disc height
SL: segmental lordosis
BMI: body mass index
VAS: visual analogue scale
ODI: Oswestry disability index
DDD: disc degenerative diseases
ASD: adjacent segment disease
FV: facet joint violation
LSS: lumbar spinal stenosis
LDH: lumbar disc herniation.
